# Prevalence of anal fistulas: a systematic review and meta-analysis 

**Published:** 2022

**Authors:** Arash Sarveazad, Mansour Bahardoust, Jebreil Shamseddin, Mahmoud Yousefifard

**Affiliations:** 1 *Colorectal Research Center, Iran University of Medical Sciences, Tehran, Iran*; 2 *Nursing Care Research Center, Iran University of Medical Sciences, Tehran, Iran*; 3 *Department of Epidemiology, School of Public Health, Shahid Beheshti University of Medical Sciences, Tehran, Iran*; 4 *Infectious and Tropical Diseases Research Center, Hormozgan Health Institute, Hormozgan University of Medical Sciences, Bandar Abbas, Iran*; 5 *Physiology Research Center, Iran University of Medical Sciences, Tehran, Iran*

**Keywords:** Anal Fistulas, Prevalence, Systematic Review.

## Abstract

Anal fistula refers to a clinical condition with local pain and inflammation associated with purulent discharge that affects the quality of life. Due to the lack of studies, the presence of bias, and high heterogeneity in the studies, the present systematic review is the first to be performed on the population-based database in this field. The present systematic review and meta-analysis was performed according to MOOSE guidelines. After systematic searching in electronic databases, only four articles met the inclusion criteria. After preparing a checklist and extracting data from the relevant articles, a meta-analysis was performed. All studies on the prevalence of anal fistula are related to Europe, and so far, no study has been conducted on other continents. The overall prevalence of anal fistula in European countries was 18.37 (95% CI: 18.20-18.55%) per 100,000 individuals, and the highest prevalence was reported for Italy (23.20 (95% CI: 22.82 to 23.59) per 100,000 people). From the present population-based (224,097,362) study results, it can be concluded that there is a prominent knowledge gap in this context. Because all the studies included in the current study relate only to Europe, the need for further research in this field in other countries is inevitably sensible.

## Introduction

 Anal fistula refers to a clinical condition with local pain and inflammation associated with purulent discharge that affects the quality of life (1). Most perineal abscesses originate in one of the anal glands. Obstruction of these glands causes bacterial overgrowth and, eventually, abscesses that settle in the space between the sphincters (external and internal anal sphincter) (2). These abscesses go through several exit routes. Most of them descend to the anoderm (perineal abscesses) or pass through the thickness of the external anal sphincter and end up in the ischiorectal space. A small number end up in the supralevator space. When an abscess is drained, either spontaneously or surgically, the infection's drainage route remains and is lined with epithelial tissue, leading to the formation of anal fistulas. About 60% of these abscesses lead to such a fate (3). Finally, a granular duct forms between the anorectal region and the perineal region. 1.2 to 2.8 of every 10,000 people develop anal fistula (4). A typical fistula consists of a primary (internal) and a secondary (external) orifice. Still, in some cases, the duct may become blocked along its path and a sinus remain, so perineal sinuses should be considered as a type of perinatal fistula (5). Anal fistula treatment currently includes a combination of medication and surgery to control pain, prevent secondary infections and discharge, and improve the patient's quality of life (6). Epidemiological data on the prevalence of anal fistula is essential for optimal policy-making to achieve the most effective scientific methods for controlling and treating this problem. Few studies have been done on the prevalence of anal fistula in different populations globally, and the results of these few studies are contradictory. The small number of studies, the presence of bias, and the high heterogeneity in the studies prevent scientists from providing a definitive conclusion regarding the prevalence of anal fistula. Therefore, this study aimed to investigate the prevalence of anal fistulas through a systematic review and meta-analysis approach. 

**Table 1.** Key questions and NHLBI quality control tool items

1. Was the research question or objective in this paper clearly stated?
2. Was the study population specified and defined?
3. Was the participation rate of eligible persons at least 50%?
4. Were all the subjects selected or recruited from the same or similar populations (including the same period)? Were inclusion and exclusion criteria for being in the study prespecified and applied uniformly to all participants?
5. Was a sample size justification, power description, or variance and effect estimates provided?
6. For the analyses in this paper, were the exposure(s) of interest measured before the outcome(s) being measured?
7. Was the timeframe sufficient so that one could reasonably expect to see an association between exposure and outcome if it existed?
8. For exposures that can vary in amount or level, did the study examine different exposure levels related to the outcome (e.g., categories of exposure or exposure measured as a continuous variable)?
9. Were the exposure measures (independent variables) clearly defined, valid, reliable, and implemented consistently across all study participants?
10. Was the exposure(s) assessed more than once over time?
11. Were the outcome measures (dependent variables) clearly defined, valid, reliable, and implemented consistently across all study participants?
12. Were the outcome assessors blinded to the exposure status of participants?
13. Was the loss to follow-up after baseline 20% or less?
14. Were key potential confounding variables measured and adjusted statistically for their impact on the relationship between exposure(s) and outcome(s)?

## Methods

**Study design**

The present meta-analysis was designed to summarize studies conducted on humans to evaluate the prevalence of anal fistula using the MOOSE guidelines for systematic review and meta-analysis in observational research (7). An extensive search of the electronic databases Medline, Embase, EBSCO, CINHAL, Scopus, and Web of Sciences was conducted by the end of June 2020.

**PICO definition**

The problem or population (P) of the study was human society, factor (I) was anal fistula, and the outcome (O) was the prevalence of anal fistula. Comparison (C) was not applicable in this study.

**Search strategy**

To achieve the present study’s objectives, an extensive search of electronic databases and the references of related articles was conducted. A gray literature search was also done in the present study. The search strategy was based on keywords related to anal fistula, and the process of how to search and summarize data has been reported in previous meta-analyses (8-16). The Medline (PubMed) search strategy as a template is as follows:

1. "Rectal Fistula”(Mesh) OR Rectal Fistula(tiab) OR Fistula, Rectal(tiab) OR Anal Fistula(tiab) OR anus fistula(tiab) OR anal fistula(tiab) OR anal fistule(tiab) OR fistula ani(tiab) OR perianal fistula(tiab) OR Fistula-in-ano(tiab)
2. “Prevalence”(Mesh) OR “Epidemiology”(Mesh) OR “Incidence”(Mesh) OR Prevalence(tiab) OR Epidemiology(tiab) OR Incidence(tiab) OR incidence rate(tiab) OR rate, incidence(tiab) OR epidemiologic(tiab)
3. #1 AND #2

**Inclusion and exclusion criteria**

The present study's inclusion criteria included observational studies performed on a human population without age, sex, or racial restrictions. Analyses performed on the general population were included. The prevalence of anal fistula was reported in positive cases in a specific population (for example, 10,000 people). Any study performed in a clinic or hospital or on a particular disease, such as Crohn's disease, trauma, postoperative fistula, and postpartum fistula, was excluded, because the prevalence reported in these studies was falsely high and did not provide actual data on the prevalence of this problem in the general community. Reviews and case reports were also excluded.

**Data Extraction**

Two independent researchers collected the data. After combining the results from the databases in the Endnote program and performing the gray literature search (Google search and Google Scholar), the two researchers performed initial screenings independently. The title and abstract of each article were studied, and if the article was relevant or likely to be related, the full text of the article was reviewed. ’The extracted data was then summarized in a checklist designed based on the PRISMA statement guidelines (17) and included information related to the study design, sample characteristics, the number of samples studied, data collection period, country studied, the population covered in the study, age range, and the number of anal fistula cases.

**Quality control of the articles**

Articles were controlled for quality according to NHLBI guidelines (18), a tool containing 14 items for examining the quality of observational articles. Based on the 14 key questions presented below (Table 1), each item received a score of Yes / No / Not applicable / Not reported / cannot be determined. A “YES” score was awarded when the item in the question was clearly and thoroughly explained in the article. If the question was not answered in the article, a “NO“ was scored. If no information about the item was reported in the article, it received a “Not reported score," and if it was not possible to determine an answer based on the available information in the article, a score of “Could not be determined” was assigned. If the item was not applicable to the study, the answer was“Not applicable."

**Statistical analysis**

Analysis was performed using STATA 14.0 statistical program. All studies were summarized based on the studied outcome, the prevalence of anal fistula in the general population. As some studies reported the number of cases of anal fistula in 10,000 people and some studies reported cases in 100,000 people, the number of cases was reported in 100,000 patients to conviniently perform the meta-analysis. The presence of heterogeneity was investigated using the I^2^ test. Based on the presence or absence of heterogeneity, a random effect model or a fixed-effect model was used to perform the analysis, respectively. Beg’s test and Funnel Plot were used to identify publication bias. 

## Results

**Characteristics of the included studies**

**Figure 1:**
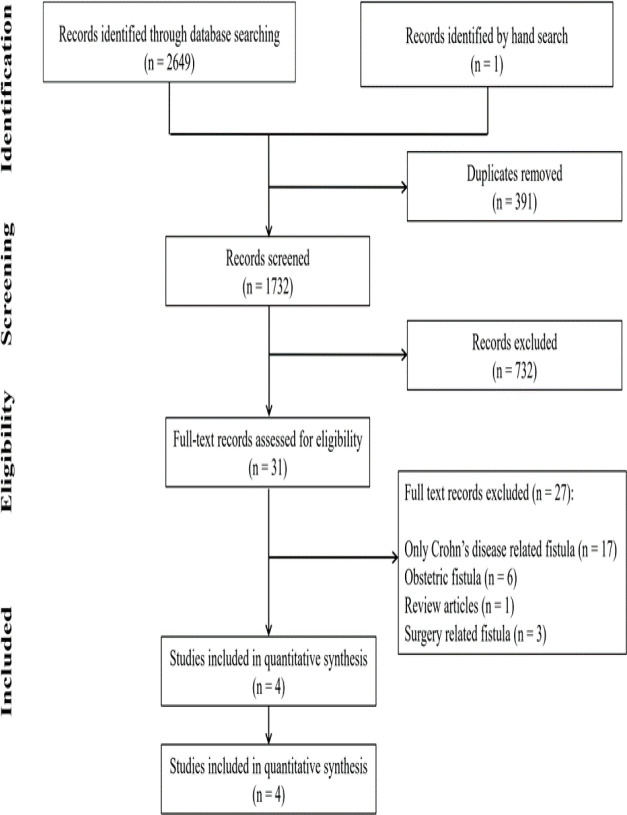
Flowchart of the present study

The initial search resulted in 2649 studies. After eliminating duplicates and reading the titles and abstracts of the remaining articles, the full texts of 31 articles were studied in more detail. Eventually, 27 studies were excluded, 17 due to evaluation of the prevalence of anal fistula in patients with Crohn's disease, six due to the prevalence of anal fistula after parturition, three due to the prevalence of anal fistula after surgery, and one for being a review article. Figure 1 shows the selection process of articles in the present study. 

The four studies included were all conducted in Europe (4, 19-21). The first study was conducted in Finland between 1969 and 1978. The second study was a 30-center study in continental Europe conducted between 1980 and 1994. The third study was a quadrilateral study conducted in Spain (2001), Germany (2002), Italy (2002), and the United Kingdom (2004). 

**Table 2:** Characteristics of the entered studies

Study	Recruitment period	Country	Population coverage*	Study design	Age group	Male number	Number of fistulae
Cuschieri; 2001	1980-1994	European countries	4.60	Registry survey	Newborns	NR	72
							359
							47
Hokkanen; 2019	2014	UK	4.88	Registry survey	Children and adult	NR	1143
	2015	UK	4.22				938
	2016	UK	3.57				688
	2017	UK	3.17				579
Sainio; 1984	1969-1978	Finland	0.51	Retrospective analysis	Children and adult	288	458
							
Zanotti; 2007	2004	England	49.56	Registry survey	Children and adult	NR	9104
	2002	Germany	82.54				16645
	2002	Italy	56.99				13231
	2001	Spin	14.05				1458

*, Numbers are presented in a million population

**Table 3:** Quality control of imported articles

Item	Cuschieri; 2001	Hokkanen; 2019	Sainio; 1984	Zanotti; 2007
1. Was the research question or objective in this paper clearly stated?	Yes	Yes	Yes	Yes
2. Was the study population specified and defined?	Yes	Yes	Yes	Yes
3. Was the participation rate of eligible persons at least 50%?	Yes	Yes	NR	Yes
4. Were all the subjects selected or recruited from the same or similar populations (including the same period)? Were inclusion and exclusion criteria for being in the study prespecified and applied uniformly to all participants?	Yes	Yes	Yes	Yes
5. Was a sample size justification, power description, or variance and effect estimates provided?	No	No	No	No
6. For the analyses in this paper, were the exposure(s) of interest measured before the outcome(s) being measured?	NA	NA	NA	NA
7. Was the timeframe sufficient so that one could reasonably expect to see an association between exposure and outcome if it existed?	Yes	Yes	Yes	Yes
8. For exposures that can vary in amount or level, did the study examine different exposure levels related to the outcome (e.g., categories of exposure or exposure measured as a continuous variable)?	NA	NA	NA	NA
9. Were the exposure measures (independent variables) clearly defined, valid, reliable, and implemented consistently across all study participants?	Yes	Yes	Yes	Yes
10. Was the exposure(s) assessed more than once over time?	NA	NA	NA	NA
11. Were the outcome measures (dependent variables) clearly defined, valid, reliable, and implemented consistently across all study participants?	Yes	Yes	Yes	Yes
12. Were the outcome assessors blinded to the exposure status of participants?	No	No	No	No
13. Was the loss to follow-up after baseline 20% or less?	NR	NR	NR	NR
14. Were key potential confounding variables measured and adjusted statistically for their impact on the relationship between exposure(s) and outcome(s)?	NA	NA	NA	NA

NA: Not applicable; NR: Not reported

The fourth article contained data collected in the UK in 2014, 2015, 2016, and 2017. In the study of Cuschieri et al. in Europe, the prevalence of anal fistula was reported by three etiologies. Therefore, out of four studies, 12 separate experiments were entered in the meta-analysis. These population-based studies represented 224,097,362 members of the general public. The number of patients with anal fistula in the comprehensive study was 44,722. f 2 shows the details of the imported articles.

**Quality control**

The quality of the articles was controlled according to the recommended guidelines of NHLBI (Table 3). None of the studies reported the method of determining the sample size; however, because the number of samples entered was very large and covered a significant population, the researchers determinedthat this factor did not affect the quality of these four studies. The prevalence of anal fistula was not evaluated in any of the blinded studies. Lack of blindness also did not affect these studies' findings, because the report of anal fistula prevalence was mainly based on registry databases. The blindness of the researcher did not affect the classification of individuals as patients or the patients either. None of the four studies reported a drop in patients. The reason for this lack of reporting was the retrospective nature of these studies. Because missing data is commonly mentioned in registry databases, the researchers in these four studies could point out that this is a negative point. As the condition of blinding and determining the sample size did not significantly affect the prevalence of anal fistula, generally, the quality of the articles is in a good range.

**Publication bias**

**Figure 2:**
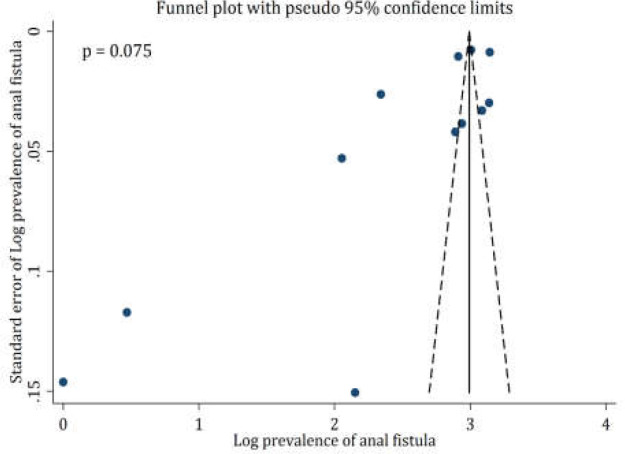
Publication bias between included studies

Begg’s test and funnel plot were used to determine the possible publication bias. Although the Begg’s test showed no publication bias in the present study (*p* = 0.075), the funnel plot indicated there is a possibility of publication bias (Figure 2). This may be because all of the included studies were related to Europe, and no population-based study in other parts of the world was found. Therefore, the researchers of the present study consider the possibility of publication bias.

**Prevalence of anal fistula**

**Figure 3:**
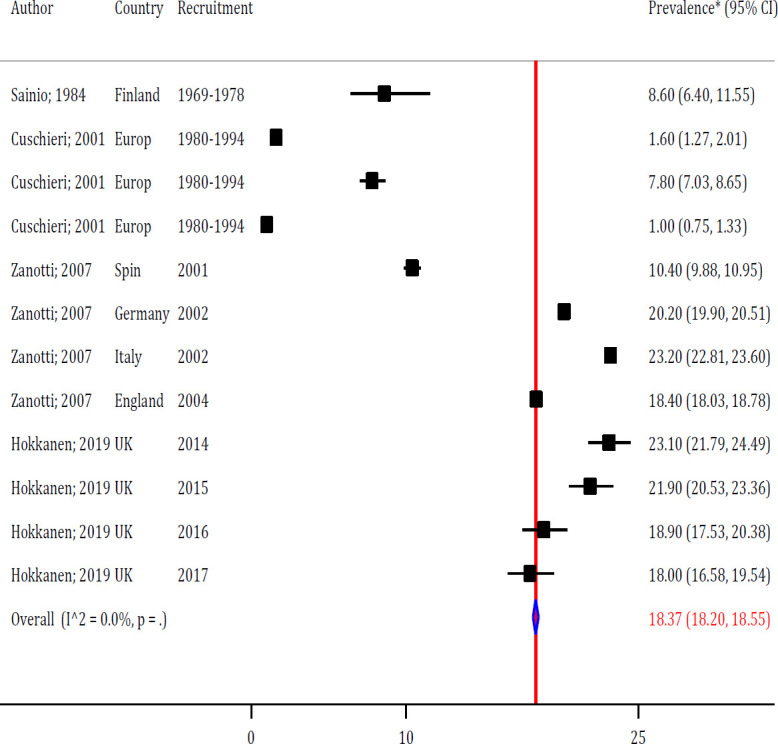
Forest plot the overall prevalence of anal fistula in the general population

**Figure 4:**
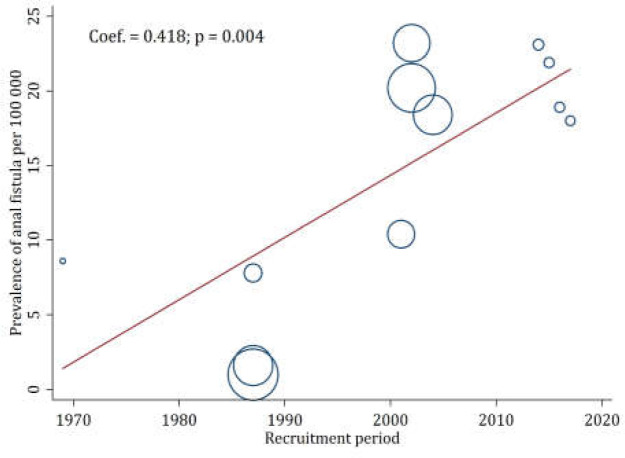
Prevalence of anal fistula by year of data collection

The overall prevalence of anal fistula in European countries was 18.37 (95% CI: 18.20-18.55%) per 100,000 individuals (Figure 3). No heterogeneity was observed between studies (I2 = 0.0%). As shown in Figure 3, the prevalence of anal fistula in the years before 2000 is much lower than in the years after 2000. Therefore, by performing metaregression, it was found that the year of the study (sampling year) is the main cause of heterogeneity between studies (meta-regression coefficient = 0.418; *p* = 0.004) (Figure 4).

* Prevalence has been reported in the number of cases per 100,000 general population.

**Prevalence of anal fistula by country studied**

**Figure 5:**
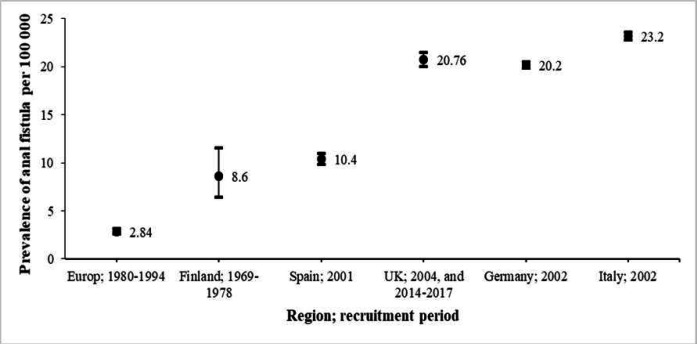
Prevalence of anal fistula by study countries. The prevalence is estimated as the number of patients per 100,000 population. Data are reported to be prevalent with a 95% confidence interval

Figure 5 shows the prevalence of anal fistula in different countries. The highest prevalence was reported for Italy (2002, one study) as 23.20 (95% CI: 22.82 to 23.59) per 100,000 people. The prevalence rates of anal fistula in Germany (2002; one study), United Kingdom (2004 and 2014-2017; 5 studies), Spain (2001, one study), and Finland (1969 to 1978; one study) are shown separately in Table 4.

## Discussion

Because epidemiological data on the prevalence of anal fistula is essential to adopting the best strategy for the management and treatment of this complication, for the first time, in this study, a systematic review was performed on the population-based databases in this field. One of the most important findings of the present meta-analysis is that all studies on the prevalence of anal fistula are related to Europe. To date, no study has been conducted in other continents, and this meta-analysis revealed a large knowledge gap in this context. The current findings showed that among 224,097,362 people (the total number of people studied in the existing studies), there were 44,722 cases of anal fistula, and the total prevalence of anal fistula in European countries is 12.81 people per 100,000 people. The highest prevalence of anal fistula was found in Italy. Other systematic reviews in this area, such as the 2019 study by Garcıa-a-Olmo et al., have examined studies showing the prevalence of anal fistula in a specific population, such as patients with Crohn's syndrome or trauma (treatment and surgery complications). Their study reported incidence of anal fistula in cryptoglandular infection, trauma, and Crohn's disease is 1.69 per 10,000 people (22). However, the present meta-analysis is the only population-based study that has examined the prevalence of anal fistulas in the general population (not a specific group). One of this study's strengths is its population-based nature, because in epidemiological studies, this component is considered to be the gold standard. Therefore, the systematic review and population-based nature of our study significantly increase the validity of the present study's findings. 

One of the limitations of the present study is the lack of existing studies in this field. As there are only four studies in this field, all of which are related to Europe, it is strongly recommended that the prevalence of anal fistula in other parts of the world be investigated to achieve a complete database. Due to the lack of epidemiological studies in this area, the presence of heterogeneity and publication bias was predictable. Also, because of the retrospective nature of the articles and extraction of data from registry sources with high sample sizes, the lack of blindness and lack of reporting on the method of determining sample size can be ignored. It can be said that heterogeneity is not related to the articles’ quality. Meta-translation in the current study showed that the origin of heterogeneity is the year of study (sampling year), as the prevalence of anal fistula in the years before 2000 is much lower than in the years after 2000. Although being population-based is one of the strengths of epidemiological studies, including the present one, such data sources are always likely to be biased because of inadequate classification or incomplete reporting of diagnostic procedures and processes.

From the present results, it can be estimated that the prevalence of anal fistula in the general European population (not a specific population with a specific disease or complication) is equal to 18.37 per 100,000 people, and the highest prevalence is in Italy. The validity of the present results can be confirmed in part by the population-based nature of this study (224,097,362). The lack of similar data in other parts of the world indicates the urgent need for similar studies in other countries.

**Table 4:** Prevalence of anal fistula by study countries

Region and recruitment period	Prevalence	95% confidence interval	
European countries; 1980-1994	2.84	2.57	3.13
Finland; 1969-1978	8.6	6.4	11.55
Spain; 2001	10.4	9.88	10.95
UK; 2004, and 2014-2017	20.76	20.06	21.47
Germany; 2002	20.2	19.9	20.51
Italy; 2002	23.2	22.82	23.6

The prevalence is estimated as the number of patients per 100,000 population

## Conflict of interests

The authors declare that they have no conflict of interest.
